# COVID-19 Home Monitoring After Diagnosis and Health Care Utilization in an Integrated Health System

**DOI:** 10.1001/jamahealthforum.2021.0333

**Published:** 2021-05-06

**Authors:** Anita D. Misra-Hebert, Xinge Ji, Lara Jehi, Alex Milinovich, Elizabeth R. Pfoh, Michael W. Kattan, James B. Young

**Affiliations:** 1 Healthcare Delivery and Implementation Science Center, Cleveland Clinic, Cleveland, Ohio; 2 Department of Quantitative Health Sciences, Cleveland Clinic, Cleveland, Ohio; 3 Department of Neurology, Neurological Institute, Cleveland Clinic, Cleveland, Ohio; 4 Center for Value-Based Care Research, Cleveland Clinic Community Care, Cleveland, Ohio

## Abstract

This cohort study examines health care utilization patterns for patients with COVID-19 who were enrolled vs not enrolled in a home monitoring program.

## Introduction

Remote monitoring programs have been implemented for patients with suspected or confirmed COVID-19^1^ after hospital discharge^2,3^ or emergency department (ED) visits.^4^ The Cleveland Clinic Health System (CCHS) established a home monitoring program (HMP) for patients with positive test results for SARS-Co-V-2. We assessed health care utilization patterns for patients enrolled in the HMP compared with similar patients who were not enrolled.

## Methods

We identified patients with positive test results for SARS-CO-V-2 in the CCHS (US Centers for Disease Control and Prevention assay, Roche magnapure extraction, ABI 7500 DX polymerase chain reaction) from March 1 to July 31, 2020, from the CCHS COVID-19 registry, which included demographic and clinical variables. Utilization in the year before the SARS-Co-V-2 test (eg, hospitalizations, ED, outpatient visits) was obtained from the electronic medical record (EMR). While all patients with COVID-19 were offered HMP enrollment to receive telephone outreach (a description of the HMP can be found in eAppendix 1 in the Supplement), to more confidently capture EMR utilization data, we limited our analysis to patients with an assigned CCHS primary care physician (the study population is described in eAppendix 2 in the Supplement). Race/ethnicity was captured from the EMR and included, given its potential contribution to COVID-19 outcomes. Descriptive statistics were reported as counts (percentages) or median values (interquartile ranges). For demographic characteristic and comorbidity comparisons, Wilcoxon signed-rank tests were used for numeric variables and χ^2^ or Fisher exact tests for categorical variables. Overlap propensity score weighting^5^ using all collected variables was then used to address baseline group differences in those enrolled vs not enrolled in the HMP. The 30-day and 90-day utilization outcomes, including hospitalizations (primary outcome) and ED and outpatient visits, were measured while excluding ED visits or hospitalizations that occurred within 24 hours of the positive SARS-Co-V-2 test result. Subgroup analyses were performed for patients with positive test results that were associated with hospitalization/ED or outpatient visits. This study was approved by the CCHS institutional review board as minimal risk; thus, consent was not required. The reporting follows the Strengthening the Reporting of Observational Studies in Epidemiology (STROBE) reporting guideline. All statistical analyses were performed using the tidyverse and survey package with R software (R Foundation). Statistical significance was set at *P *< .05.

## Results

Baseline characteristics of the study population by HMP participation and subgroup are shown in Table 1. There were 3975 patients who participated and 3221 who did not. Those participating overall were younger; more likely to be women, Black individuals, and/or individuals with non-Hispanic ethnicity; and have more asthma diagnoses, a lower proportion of several other comorbidities, and more prior year outpatient visits. Overlap propensity score weighting and odds ratios (ORs) for the outcomes are shown in Table 2. There were lower odds of 30-day or 90-day hospitalization (OR, 0.73; 95% CI, 0.60-0.88; and OR, 0.79; 95% CI, 0.67-0.93, respectively) but no significant association of the HMP with 30-day or 90-day ED utilization (OR, 0.91; 95% CI, 0.75-1.12; and OR, 0.96; 95% CI, 0.81-1.15, respectively), and there were higher odds of outpatient visits at 30 and 90 days (OR, 1.97; 95% CI, 1.68-2.30; and OR, 2.09; 95% CI, 1.76-2.48, respectively). A subgroup analysis showed lower odds of future hospitalization that was limited to patients posthospitalization or ED visits (OR, 0.62; 95% CI, 0.48-0.81; and OR, 0.70; 95% CI, 0.55-0.89 for 30-day and 90-day hospitalization, respectively).

**Table 1.** Patients With Positive SARS-CoV-2 Test Result by Home Monitoring Program Participation (Overall and Subgroups)

Characteristic	Overall			Outpatient			Emergency department/hospitalization		
	Positive test result, No. (%)		*P* value	Positive test result, No. (%)		*P* value	Positive test result, No. (%)		*P* value
	Home monitoring, no	Home monitoring, yes		Home monitoring, no	Home monitoring, yes		Home monitoring, no	Home monitoring, yes	
No.	3221	3975	NA	1950	2672	NA	1271	1303	NA
Age, median (IQR), y	57.3 (38.9-72.6)	48.1 (31.7-60.4)	<.001	57.6 (40.0-73.8)	45.0 (30.2-58.1)	<.001	56.5 (37.3-70.9)	52.9 (37.0-64.1)	<.001
Sex									
Male	1389 (43.1)	1595 (40.1)	.01	735 (37.7)	996 (37.3)	.80	654 (51.5)	599 (46.0)	.01
Female	1832 (56.9)	2380 (59.9)		1215 (62.3)	1676 (62.7)		617 (48.5)	704 (54.0)	
Race									
Black	856 (26.6)	1495 (37.6)	<.001	358 (18.4)	856 (32.0)	<.001	498 (39.2)	639 (49.0)	<.001
White	2034 (63.1)	2047 (51.5)		1387 (71.1)	1534 (57.4)		647 (50.9)	513 (39.4)	
Other	331 (10.3)	433 (10.9)		205 (10.5)	282 (10.6)		126 (9.9)	151 (11.6)	
Missing	154 (4.8)	187 (4.7)		106 (5.4)	140 (5.2)		48 (3.8)	47 (3.6)	
Ethnicity									
Hispanic	501 (15.6)	304 (7.6)	<.001	311 (15.9)	191 (7.1)	<.001	190 (14.9)	113 (8.7)	<.001
Non-Hispanic	2561 (79.5)	3486 (87.7)		1525 (78.2)	2331 (87.2)		1036 (81.5)	1155 (88.6)	
Unknown	159 (4.9)	185 (4.7)		114 (5.8)	150 (5.6)		45 (3.5)	35 (2.7)	
Comorbidities									
Asthma	453 (14.1)	784 (19.7)	<.001	264 (13.5)	497 (18.6)	<.001	189 (14.9)	287 (22.0)	<.001
COPD/emphysema	286 (8.9)	200 (5.0)	<.001	164 (8.4)	75 (2.8)	<.001	122 (9.6)	125 (9.6)	>.99
Diabetes	707 (21.9)	699 (17.6)	<.001	386 (19.8)	378 (14.1)	<.001	321 (25.3)	321 (24.6)	.75
Hypertension	1567 (48.6)	1569 (39.5)	<.001	940 (48.2)	928 (34.7)	<.001	627 (49.3)	641 (49.2)	.98
Coronary artery disease	465 (14.4)	272 (6.8)	<.001	283 (14.5)	138 (5.2)	<.001	182 (14.3)	134 (10.3)	.002
Heart failure	387 (12.0)	198 (5.0)	<.001	227 (11.6)	78 (2.9)	<.001	160 (12.6)	120 (9.2)	.01
Cancer	434 (13.5)	376 (9.5)	<.001	276 (14.2)	256 (9.6)	<.001	158 (12.4)	120 (9.2)	.01
Transplant history	36 (1.1)	27 (0.7)	.06	14 (0.7)	17 (0.6)	.88	22 (1.7)	10 (0.8)	.04
Multiple sclerosis	37 (1.1)	26 (0.7)	.04	18 (0.9)	19 (0.7)	.53	19 (1.5)	7 (0.5)	.03
Connective tissue disease	174 (5.4)	225 (5.7)	.67	108 (5.5)	133 (5.0)	.44	66 (5.2)	92 (7.1)	.06
Inflammatory bowel disease	92 (2.9)	106 (2.7)	.68	50 (2.6)	72 (2.7)	.86	42 (3.3)	34 (2.6)	.36
Immuno-suppressive disease	454 (14.1)	372 (9.4)	<.001	255 (13.1)	195 (7.3)	<.001	199 (15.7)	177 (13.6)	.15
**Previous utilization**									
Emergency department in previous year									
Median (IQR)	1.0 (0-2.0)	1.0 (0-2.0)	<.001	0 (0-0)	0 (0-2.0)	<.001	2.0 (1.0-4.0)	2.0 (1.0-4.0)	.16
Mean (SD)	1.6 (2.9)	1.7 (2.7)	.06	0.7 (2.0)	1.1 (2.2)	<.001	3.0 (3.5)	2.9 (3.2)	.66
Outpatient in previous year									
Median (IQR)	4.0 (0-13.0)	9.0 (3.0-18.0)	<.001	4.0 (1.0-12.8)	8.0 (3.0-18.0)	<.001	3.0 (0-12.0)	6.0 (1.0-18.0)	<.001
Mean (SD)	10.7 (19.2)	14.3 (19.2)	<.001	10.2 (17.2)	13.8 (18.5)	<.001	11.3 (21.9)	13.4 (20.2)	.01
Hospitalization in previous year									
Median (IQR)	1.0 (0-3.0)	1.0 (0-3.0)	.18	0 (0-1.0)	1.0 (0-2.0)	<.001	2.0 (1.0-4.0)	2.0 (1.0-4.0)	.24
Mean (SD)	2.1 (3.4)	1.2 (2.8)	.17	1.1 (2.2)	1.3 (2.2)	.001	3.6 (4.1)	3.3 (3.5)	.09

Abbreviations: COPD, chronic obstructive pulmonary disease; IQR, interquartile range.

**Table 2.** Patients With Positive SARS-CoV-2 Test Result by Home Monitoring Program Participation and the Overlap Between Propensity Score Weighted Characteristics and Outcomes Overall and by Subgroup

Characteristic	Overall		Outpatient		Emergency department/hospitalization	
	Home monitoring, no	Home monitoring, yes	Home monitoring, no	Home monitoring, yes	Home monitoring, no	Home monitoring, yes
Count, No.	3221	3975	1950	2672	1271	1303
Age, y^a^	50.8	50.8	50.0	50.0	52.7	52.7
Sex						
Men	42.6	42.6	38.3	38.3	49.0	49.0
Women	57.4	57.4	61.7	61.7	51.0	51.0
Race						
Black	32.0	32.0	24.0	24.0	44.3	44.3
White	57.1	57.1	65.1	65.1	44.6	44.6
Other^b^	10.9	10.9	10.9	10.9	11.1	11.1
Ethnicity						
Hispanic	11.4	11.4	12.0	12.0	11.0	11.0
Non-Hispanic	83.8	83.8	82.4	82.4	85.8	85.8
Unknown	4.8	4.8	5.6	5.6	3.2	3.2
Asthma	16.3	16.3	15.2	15.2	17.8	17.8
COPD/emphysema	6.5	6.5	4.5	4.5	9.4	9.4
Diabetes	19.2	19.2	16.2	16.2	24.6	24.6
Hypertension	43.1	43.1	40.1	40.1	48.6	48.6
Coronary artery disease	9.5	9.5	8.1	8.1	11.8	11.8
Heart failure	7.2	7.2	5.1	5.1	10.4	10.4
Cancer	11.2	11.2	11.7	11.7	10.3	10.3
Transplant history	0.9	0.9	0.7	0.7	1.1	1.1
Multiple sclerosis	0.8	0.8	0.9	0.9	0.8	0.8
Immunosuppressive disease	11	11	8.9	8.9	14	14
Connective tissue disease	5.3	5.3	4.8	4.8	5.9	5.9
Inflammatory bowel disease	2.8	2.8	2.6	2.6	3.0	3.0
Emergency department in previous year	1.6	1.6	0.8	0.8	2.9	2.9
Outpatient visit in previous year	12.4	12.4	12.2	12.2	12.3	12.3
Hospitalization in previous year	2.0	2.0	1.1	1.1	3.4	3.4
**Outcome**						
Emergency department						
30 d	14.5	13.4	9.9	10.2	21.4	18.9
OR (95% CI)		0.91 (0.75-1.12)		1.03 (0.76-1.39)		0.85 (0.64-1.13)
90 d	20.0	19.4	14.1	14.3	28.9	27.7
OR (95% CI)		0.96 (0.81-1.15)		1.01 (0.78-1.31)		0.94 (0.73-1.21)
Outpatient						
30 d	63.1	77.1	71.1	78.8	53.3	74.4
OR (95% CI)		1.97 (1.68-2.30)		1.51 (1.23-1.87)		2.55 (2.00-3.25)
90 d	71.2	83.8	79.2	85.4	61.7	80.8
OR (95% CI)		2.09 (1.76-2.48)		1.54 (1.21-1.96)		2.62 (2.02-3.40)
Hospitalization						
30 d	18.7	14.4	12.4	11.3	28.7	20.0
OR (95% CI)		0.73 (0.60-0.88)		0.90 (0.68-1.20)		0.62 (0.48-0.81)
90 d	25.3	21.1	17.7	16.3	37.2	29.4
OR (95% CI)		0.79 (0.67-0.93)		0.91 (0.71-1.15)		0.70 (0.55-0.89)

Abbreviations: COPD, chronic obstructive pulmonary disease; OR, odds ratio.

^a^ Reported are either weighted proportions (for categorical variables) or weighted means (for numeric variables).

^b^ The Other category includes race other than Black or White, including Asian, Native Hawaiian or other Pacific Islander, American Indian or Alaska Native, multiracial, and missing/unknown.

## Discussion

The COVID-19 HMP was associated with lower odds of hospitalization, particularly for the posthospital or ED subgroup, with no significant association with ED utilization up to 90 days after diagnosis and with higher odds of subsequent outpatient utilization. Limitations include that these findings are from a single health system, and our analytic methods may not have adjusted for all confounders, specifically the choice for HMP enrollment. The COVID-19 pandemic has prompted resource distribution discussions and concern for missed opportunities for chronic disease management.^6^ This study’s outcomes support the need for randomized clinical trials to evaluate HMPs and consideration of targeted resource allocation for home monitoring after COVID-19 diagnosis or to other opportunities to maintain the health of patients during the pandemic.

## Notes

Supplement. eAppendix 1. Additional description of COVID-19 home monitoring program eAppendix 2. Creation of study population
