# Supplementary material for: COVID-19 Home Monitoring After Diagnosis and Health Care Utilization in an Integrated Health System
Source: JAMA Health Forum. 2021 May 6;2(5):e210333. doi: 10.1001/jamahealthforum.2021.0333 (PMC8796892; doi:10.1001/jamahealthforum.2021.0333)
Supplement: Supplement. — eAppendix 1. Additional description of COVID-19 home monitoring program eAppendix 2. Creation of study population [file jamahealthforum-e210333-s001.pdf]

Supplemental Online Content

Misra-Hebert AD, Ji X, Jehi L, et al. COVID-19 home monitoring after diagnosis and health care utilization in an integrated health system. *JAMA Health Forum*. Published online May 6, 2021. doi:10.1001/jamahealthforum.2021.0333

eAppendix 1. Additional description of COVID-19 home monitoring program

eAppendix 2. Creation of study population

This supplemental material has been provided by the authors to give readers additional information about their work.

© 2021 Misra-Hebert AD et al. *JAMA Health Forum.*

eAppendix 1: Methods-Additional Description of COVID-19 Home Monitoring Program

The Cleveland Clinic Health System (CCHS) COVID-19 home monitoring program is delivered through our primary care operational team and during the time of this study period included daily telephone outreach to patients who tested positive for SARS-Co-V-2 infection for 10-14 days after outpatient testing or 7 days after hospital discharge. Enrollment was offered at time of notification of positive SARS-Co-V-2 result (or hospital discharge) whether the test was obtained in the hospital, emergency department, or outpatient setting via telephone, with attempts to reach a patient made over a 3 day period. The program was later expanded to include patients with suspected COVID-19 illness.

The EPIC electronic medical record My Chart Care Companion digital tool was also offered as a supplement to the telephone monitoring. The home monitoring telephone calls were provided by a variety of CCHS patient-facing clinical staff (for example, nurses, medical assistants, clinical technicians) who were trained by registered nurse team leads and personnel from the CCHS Nursing Education department. The content of the outreach call was designed to be algorithmic, and allowed for escalation to higher trained individuals when needed. If new symptoms were identified, an appointment was scheduled with a virtualist primary care physician. Updates were provided to the outreach team through a daily virtual huddle and written resource handout.

The outreach workforce utilized a standard outreach encounter and documentation template which provided scripting and standardization in outreach content. Symptoms were assessed based on their presence or absence, with cascading questions for further clarification of some positive responses. The standardized assessment questions were directly aligned with the MyChart Care Companion digital tool. Data was tracked in a “smartform” in the electronic medical record allowing visualization of symptom pattern over time.

While our reported analysis is limited to patients with a CCHS primary care physician (PCP), the program was also offered to patients without a PCP and patients with a PCP outside the health system thus did serve as a safety net in that approach.

© 2021 Misra-Hebert AD et al. *JAMA Health Forum.*

eAppendix 2: Methods-Creation of Study Population

Step 1: Identify all patients during study period with Positive SARS-CoV-2 test in Cleveland Clinic Health System COVID-19 Registry (N=10488)

Step 2: Remove patients with death during the hospital admission associated with the positive SARS-Co-V-2 test; all other patients were eligible for home monitoring program participation (N=10388)

Step 3: Limit dataset to patients with an assigned Cleveland Clinic Health System primary care physician (N=7196)

Step 4: Identify patients who participated in home monitoring program (N=3975)

Step 5: Remaining patients assigned to comparison group (N=3221)

© 2021 Misra-Hebert AD et al. *JAMA Health Forum.*
